# Prediction of Soil Water-Soluble Organic Matter by Continuous Use of Corn Biochar Using Three-Dimensional Fluorescence Spectra and Deep Learning

**DOI:** 10.1155/2023/7535594

**Published:** 2023-03-06

**Authors:** Liang jin, Dan Wei, Dawei Yin, Guoyuan Zou, Yan Li, Yitao Zhang, JianLi Ding, Lei Wang, Lina Liang, Lei Sun, Wei Wang, Huibo Shen, Yuxian Wang, Junsheng Xu

**Affiliations:** ^1^Plant Nutrition and Resources Institute, Beijing Academy of Agriculture and Forestry Sciences, Beijing100097, China; ^2^College of Agricultural Science and Technology, Heilongjiang Bayi Agricultural University, Daqing 163319, China; ^3^Institute of Geographic Sciences and Natural Resources Research, Beijing 100101, China; ^4^Heilongjiang Institute of Black Soil Protection and Utilization, Harbin 150086, China; ^5^Qiqihar Branch of Heilongjiang Academy of Agricultural Sciences, Qiqihar 161006, China; ^6^Qingdao Reserved Materials Management Station, Qingdao 266000, China

## Abstract

The purpose is to study the soil's water-soluble organic matter and improve the utilization rate of the soil layer. This exploration is based on the theories of three-dimensional fluorescence spectroscopy, deep learning, and biochar. Chernozem in Harbin City, Heilongjiang Province, is taken as the research object. Three-dimensional fluorescence spectra and a deep learning model are used to analyze the content of water-soluble organic matter in the soil layer after continuous application of corn biochar for six years and to calculate different fluorescence indexes in the whole soil depth. Among them, the three-dimensional fluorescence spectrum theory provides the detection standard for the application effect detection of biochar, the deep learning theory provides the technical support for this exploration, and the biochar theory provides the specific research direction. The results show that the application of corn biochar for six consecutive years significantly reduces the average content of water-soluble organic matter in different soil layers. Among them, the highest average content of soil water-soluble organic matter is “nitrogen, potassium, phosphorous” (NPK) and the lowest is “boron, carbon” (BC). Comparing the soil with BC alone, in the topsoil, the second section (330–380 nm/200–250 nm) with BC + NPK increases by 13.3%, the third section (380–550 nm/220–250 nm) increases by 8.4%, and the fourth section (250–380 nm/250–600 nm) increases by 50.1%. The combination of nitrogen (N) + BC has a positive effect of 20.7%, 12.2%, and 28.4% on sections I, II, and IV, respectively. In addition, in the topsoil, the combination of NPK + BC significantly increases the content of acid-like substances compared with the application of BC alone. In the black soil, with or without fertilizer NPK, there is no significant difference in the level of fulvic acid-like components. The prediction of soil water-soluble organic matter after continuous application of corn biochar based on three-dimensional fluorescence spectra and deep learning is carried out, which has reference significance for the rapid identification and early prediction of subsequent soil activity.

## 1. Introduction

With the development of society and the continuous increase of population, the social demand for agriculture is higher and higher, so it is imperative to improve agricultural productivity through various means. Among them, agricultural fertilization is the best way to promote the stable improvement of agricultural productivity, but excessive agricultural fertilization will cause irresistible harm to the land. Hence, in recent years, due to intensive agricultural management, people have conducted extensive research on the input of farmland fertilizers, especially organic materials [1]. Water-soluble organic matter can be decomposed by microorganisms and release nutrients for plants to absorb. Therefore, it is usually considered one of agricultural development's most important components. Strengthening the prediction of soil water-soluble organic matter can find the possible problems in the soil earlier and improve the yield of crops to a certain extent [2, 3].

Bradford et al. effectively integrated image technology and spectral technology in the prediction of soil water-soluble organic matter. This technology can image the object to be measured and visualize the image information of the sample. Moreover, it can obtain the spectral information of the sample to be measured under multiple continuous narrow bands and then reflect the internal main material composition through the peak and trough information. Therefore, each data acquisition obtains a hypercube generated by stacking hundreds of single-channel black-and-white (gray scale) images (each image represents the corresponding spectral wavelength band) with each other. It is a noninvasive and noncontact rapid detection technology, which can scan massive samples simultaneously, thus overcoming the limitations of conventional spectral technology that can only measure a single sample [4]. Thilakarathna and Hernandez-Ramirez established a prediction model for the content of organic matter in the soil by analyzing the relationship between indoor chemical composition and outdoor soil spectrum and applied it to hyperspectral remote sensing images to obtain the classification map of soil organic matter in this region [5]. Galicia-Andres et al. conducted plot yield and canopied spectral reflectance replication tests on three sets of soybean breeding lines at different growth stages. Hyperspectral remote sensing technology was used to predict the yield of soybean breeding areas. It was found that multiple linear regression was suitable for plot yield prediction [6]. Man et al. conducted nondestructive testing on internal defects of apples in semitransmission mode. The classification results vary with the direction of tested fiber and fruit. When using the average spectrum, better classification results can be obtained for apples with slight defects [7]. Blonska et al. studied and compared four multivariate data analysis methods to optimize beef adulteration's rapid nondestructive quantitative detection based on visible near-infrared hyperspectral imaging. The least-squares support vector machine model has achieved good prediction results, and the root mean squared errors of training and prediction are 5.39% and 6.29%, respectively [8]. Rocci et al. used perennial ryegrass as an experimental crop and combined hyperspectral imaging with machine learning to analyze the fodder value. The results show that it has a good prediction ability, which is conducive to improving the speed and reducing the cost of the commercial breeding plan [9]. Escalona et al. used hyperspectral imaging technology in the range of 400–1000 nm to predict the quality changes of corn seeds at different storage times. It mainly includes hardness, elasticity, and resilience. A partial least square regression algorithm was adopted to integrate the reference values measured by traditional methods with the extracted spectral data. The deep learning model has produced good results in predicting hardness, elasticity, and resilience. It shows that hyperspectral imaging technology can be used as a fast, accurate, and nondestructive tool to detect the impact of different storage times on the quality characteristics of corn [10].

Under this background, this exploration takes chernozem in Heilongjiang Province as the research object and applies three-dimensional fluorescence spectra and a deep learning model to analyze the content of water-soluble organic matter in the soil layer after six years of fertilization and to calculate different fluorescence indicators in the whole soil depth. The research innovation is to integrate the three-dimensional fluorescence spectrum and deep learning model and comprehensively analyze the content of water-soluble organic matter in the soil layer after continuous application of corn biochar for six years, the three-dimensional fluorescence spectrum, and different fluorescence indicators in the whole soil depth. The purpose is to provide a method for improving soil organic matter content and finding soil problems as soon as possible. It suggests that this exploration provides a reference for the optimization of biochar application and also makes a contribution to the development of agriculture in the future.

## 2. Materials and Methods

### 2.1. Three-Dimensional Fluorescence Spectroscopy and Deep Learning

**Figure 1:**
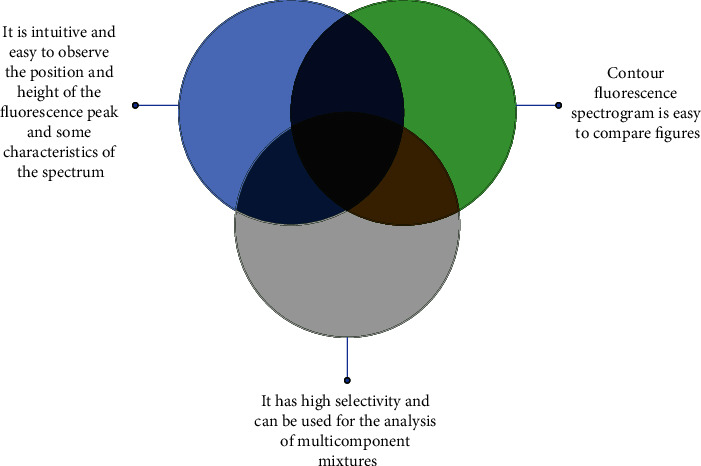
Characteristics of three-dimensional fluorescence spectra.

The three-dimensional fluorescence spectrum is an excitation-emission-matrix spectra characterized by the three-dimensional coordinates of the excitation wavelength (*y*-axis), emission wavelength (*x*-axis), and fluorescence intensity (*z*-axis), also known as the total luminescence spectrum [11]. The usual fluorescence spectrum is a plan view obtained by scanning the fluorescence intensity against the emission wavelength. The three-dimensional fluorescence spectroscopy technology can obtain the excitation wavelength, emission wavelength, and fluorescence intensity information when it changes [12]. Three-dimensional fluorescence spectrograms are generally expressed in three-dimensional projection and contour fluorescence spectrograms [13].  Figure 1 shows the characteristics of this spectrum.

Figure 1 shows that the fluorescence spectrum represented by three-dimensional projection is more intuitive, and it is easier to observe the position and height of the fluorescence peak and some characteristics of the spectrum. However, it is not easy to directly provide the information of the corresponding fluorescence emission intensity of the excitation-emission wavelength pair. In synchronous scanning with a fixed excitation wavelength and emission wavelength difference, each compound has a single fluorescence peak, and the contour map of each compound is limited to a rectangular box. If the sample contains four fluorescent compounds, there will be four rectangular boxes, but the components can be detected only in the nonoverlapping position. Contour fluorescence spectrograms make it easier to make graphic comparisons. Because the three-dimensional fluorescence spectrum has one more coordinate than the two-dimensional plan and the total fluorescence data obtained are much more than the ordinary fluorescence spectrum, it has high selectivity and can be used for the analysis of multicomponent mixtures. Based on the above characteristics, the three-dimensional fluorescence spectrum is commonly used in the following fields: (1) The contour fluorescence spectrum of blood is used to check the health of the human body. Human blood is composed of multiple components. When human blood has problems, the visible spectrum of total fluorescence will change significantly [14]. (2) The three-dimensional fluorescence spectrum helps solve criminal cases. It has a stronger discrimination ability for relevant samples. Especially, the spectral subtraction method is conducive to solving criminal cases [15].

**Figure 2:**
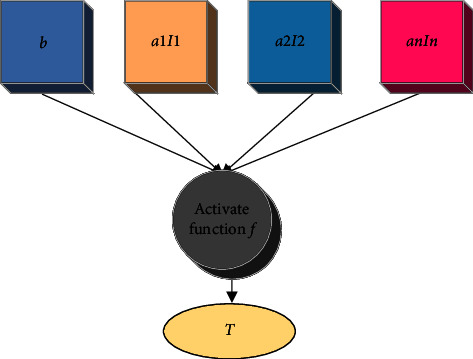
The neuronal structure.

The “deep” of deep learning is compared with the shallow machine learning method, which originates from artificial neural networks [16]. The deep neural network is the foundation of deep learning. Its structure well reflects the characteristics of “multilayer” and “nonlinear.” The neuron structure is the basic structure of the neural network system. The biological nervous system contains nervous subsystems with a different division of labor. After receiving corresponding stimulation as input, they give corresponding feedback information based on the comprehensive input results and jointly drive the normal operation of the whole system [17]. Inspired by biological neurons, artificial neurons also have a similar structure and mechanism; that is, the receiving vector is used as the input of neurons to give the corresponding results in the form of a weighted sum, and finally, the delinearization is completed through the activation function.  Figure 2 displays its basic structure.

In this model, *I*_*i*_, *i*=0,1,2 … *n* is the initial input of the model, *u* is the output of the model after linear change, and *T* is the final output of the neuron. *a*_*i*_, *i*=0,1,2 … *n* is adopted as the weight parameter of the linear transformation process of the neuron structure. *b* is the offset term, and the function *f* plays the role of delinearization [18]. Thus, equations (1) and (2) are inputs of the structure of neurons.

(1) $$\begin{array}{c} u=I^{T}A=\sum I_{i}a_{i}+b\text{,} \end{array}$$

(2) $$\begin{array}{c} T=f\left(u\right)=f\left(\sum I_{i}a_{i}+b\right)\text{,} \end{array}$$

where *f* is called the activation function.

**Figure 3:**
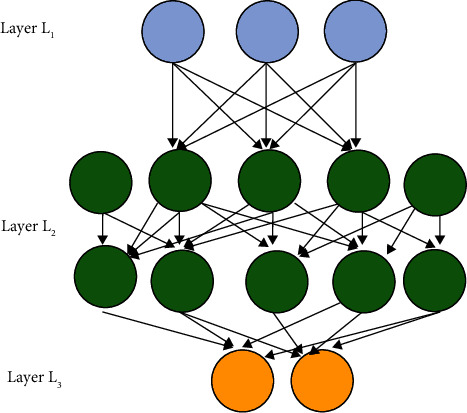
The deep learning model.

Besides, based on the neuron structure, multiple neurons are connected in turn according to different levels to form the most basic deep neural network [19]. The network model can be divided into three layers: input, hidden, and output.  Figure 3 presents the specific model.

In Figure 3, the first layer is the input layer, whose function is to input the data to be calculated into the model to provide materials for the model's operation. The second level is the hidden layer, which needs to complete the feature extraction. Each node of the hidden layer will have different receiving weights for the input information of the input layer, so it is more inclined to a certain recognition mode. That is, the meaning of the hidden layer is to abstract the characteristics of input data to another dimension of space to show its more abstract characteristics, which can be better divided linearly. Multiple hidden layers are multilevel abstractions of input features, and the ultimate goal is to better linearly divide different data types. The third level is the output layer. The function of the output layer is to classify and output the calculated data through the extracted features. It is set that the input value is *I*, the weight value of each layer is *a*, the offset term is *b*, and the activation function is *f*. The corresponding output of the hidden layer and the output layer is *T*. Equations (3)–(6) display the deduced calculation process of the forward propagation algorithm of the deep neural network.

(3) $$\begin{array}{c} T_{1}^{2}=f\left(a_{11}^{2}I_{1}+a_{12}^{2}I_{2}+a_{13}^{2}I_{3}+b_{1}^{2}\right)\text{,} \end{array}$$

(4) $$\begin{array}{c} T_{2}^{2}=f\left(a_{21}^{2}I_{1}+a_{22}^{2}I_{2}+a_{23}^{2}I_{3}+b_{2}^{2}\right)\text{,} \end{array}$$

(5) $$\begin{array}{c} T_{3}^{2}=f\left(a_{31}^{2}I_{1}+a_{32}^{2}I_{2}+a_{33}^{2}I_{3}+b_{3}^{2}\right)\text{,} \end{array}$$

(6) $$\begin{array}{c} T_{1}^{3}=f\left(a_{11}^{3}O_{1}^{2}+a_{12}^{3}O_{2}^{2}+a_{13}^{3}O_{3}^{2}+b_{1}^{3}\right)\text{.} \end{array}$$

The forward propagation process can be abstracted from the above derivation process. If there are *n* neuron structures in layer *l* − 1, equation (7) is the expression of the output of the *k*-th neuron in layer *l*.

(7) $$\begin{array}{c} T_{k}^{\prime }=f\left(\overset{n}{\underset{i=1}{\sum }}a_{ki}^{l}T_{i}^{l-1}+b_{k}^{l}\right)\text{.} \end{array}$$

The evaluation criteria of the neural network model and the corresponding optimization direction and objective are usually defined by the loss function. When the neural network is adopted to complete the classification task, the interaction entropy is usually introduced to judge the difference between the output and expected vector [20]. For given two probability distributions *p* and *q*, the interaction entropy of *p* expressed by *q* is as follows:

(8) $$\begin{array}{c} H\left(p,q\right)=-\sum p\left(x\right)\log\;q\left(x\right)\text{.} \end{array}$$

The distance between two probabilities can be described by the interaction entropy. The closer the two probabilities' distribution is, the smaller the difference between the output vector and the expected vector is, and the better the classification effect is. At this time, the value of interaction entropy will be smaller [21].

### 2.2. Biochar and Soil Water-Soluble Organic Carbon

**Figure 4:**
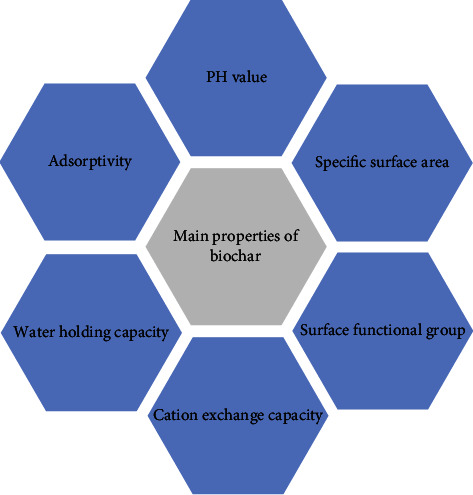
Main properties of biochar.

Biochar is a kind of charcoal as a soil conditioner, which can help plants grow. It can be used for agricultural purposes, carbon collection, and storage. It differs from the traditional charcoal commonly used for fuel [22]. In recent years, scientists have paid attention to the use of biochar due to the impact of climate change caused by the emission of greenhouse gases such as carbon dioxide, nitrous oxide, and methane. The reason is that biochar helps capture and remove greenhouse gases in the atmosphere by means of biochar storage, convert them into quite stable forms, and store them in the soil for thousands of years. In addition, biochar can increase agricultural productivity by 20%, purify water quality, and help reduce the use of chemical fertilizers [23].  Figure 4 shows its main properties.

Soil carbon pool is a crucial component of the carbon cycle and an important indicator of soil fertility and biodiversity [24]. Soil water-soluble organic carbon is one of the components of soil organic carbon, which mainly comes from fallen leaves and plant residues, microbial degradation products, and root exudates [25]. Organic manure and animal manure under human influence increase the solubility of soil organic matter, which is also one of the sources of soil water-soluble organic carbon.

The main factors affecting soil water-soluble organic carbon are as follows:

1. Soil water-soluble organic carbon is affected by multiple factors, such as climate, temperature, and vegetation coverage. Among them, the soil water-soluble organic carbon content in Phyllostachys edulis forest is the highest and that in pinus massoniana forest is the lowest. This phenomenon is related to the root system, exudates, and other factors of the vegetation [26].
2. The change of seasons will also affect soil water-soluble organic carbon. In the season with less rain, the amount of plant litter is relatively large, and the soil water-soluble organic carbon content is relatively high [27].
3. Different farming methods of human beings will lead to the loss of some soil water-soluble organic matter. According to relevant research and investigation, the large aggregates in the soil decrease significantly after 10 years of cultivation, and the water-soluble organic carbon in the surface soil also decreases [28].

## 3. Research Data Settings

Chernozem in a small town of Harbin City, Heilongjiang Province, is taken as the research object. The local long-term average annual rainfall ranges from 486.4 mm to 543.6 mm. The rainy season is mainly from June to September. The average altitude is 138 m, and the depth of groundwater level is 80 m [29, 30]. The annual average wind speed is 4.1 m/s, and the maximum wind speed is 18.9 m/s. Chernozem in a small town in Heilongjiang Province is extracted, detected, and counted based on the relevant data released by the department of ecological environment of Heilongjiang Province. Tables 1 and 2 show the local soil properties and the particle composition of biochar.

Field trials of soybean/maize rotation were conducted from 2013 to 2018. There were mainly five types of fertilization during this period, namely, carbon phosphorous (CK), nitrogen, potassium, phosphorous (NPK), 3.6 t/ha boron, carbon (BC), NPK + 3.6 t/ha BC, and nitrogen (N) + 3.6 t/ha BC. On May 14, 2013, the spring soybean variety Heinong 58 was sown and harvested on October 8. The spring maize variety Longdan 42 was sown on May 1, 2014, and harvested on October 8. From 2015 to 2018, soybeans and corn were planted in turn. The NPK fertilizer of soybean was applied before planting, while the N fertilizer of maize was applied in the form of urea. The amount of 176 kg·N/ha was used as the base fertilizer, and the remaining 50% was applied at the jointing stage [31]. As a soil conditioner, BC was applied to the ditches near the ridge every year from 2013 to 2018. The method is to thoroughly mix the soil with a plow and plow to a depth of at least 20 cm. In 2018, soil samples were collected using a soil auger (diameter of 10 cm) based on a 60 cm soil profile. Simply put, samples were taken from 0–60 cm soil layers every 10 cm and then dried at room temperature for 1 week. After sieving through a 2 mm sieve, the soil sample was kept at 4°C until the next analysis [32].

### 3.1. Extraction and Measurement of Soil Organic Matter

Soil water-soluble organic carbon was extracted by soil water oscillation. Simply put, each soil sample was mixed with deionized water at a solid water ratio of 1 : 6 (w/v) and incubated for 24 hours under continuous shaking (180 rpm). After centrifuging 10,000 g of ionic water for 6 minutes, the suspension was filtered through a cellulose acetate membrane filter (pore size: 0.45 *μ*m). After filtration, the sample was kept at −20°C for three-dimensional fluorescence spectroscopic analysis. The total organic carbon analyzer (TOC-VCPH, Shimadzu, Japan) was adopted to detect the organic carbon level in the filtrate.

The Parafac model is constructed using Matrix and Laboratory (MATLAB) 7.0 and DOMFluor toolbox to characterize the fluorescence components of soil water-soluble organic carbon. An appropriate number of components in the model are measured according to the core consistency diagnosis and the split validation test. By estimating the relative contribution of each component, the differences between different components in each sample are further compared.

Statistical Product and Service Solutions (SPSS) 20.0 (SPSS Company, Chicago, Illinois, USA) is used for data analysis. After checking homogeneity and normality, one-way ANOVA and least significant difference test are performed on the data of homogeneity variance and normal distribution. The purpose is to compare the differences in water-soluble organic matter content between various land use types and soil depths. Meanwhile, the Pearson correlation coefficient is used to determine the correlation between the parameters of water-soluble organic matter. *P* values of 0.05 and 0.01 are considered statistically significant.

## 4. Results and Discussion

### 4.1. Contents of Water-Soluble Organic Matter in Different Soil Layers after Six Years of Fertilization

**Figure 5:**
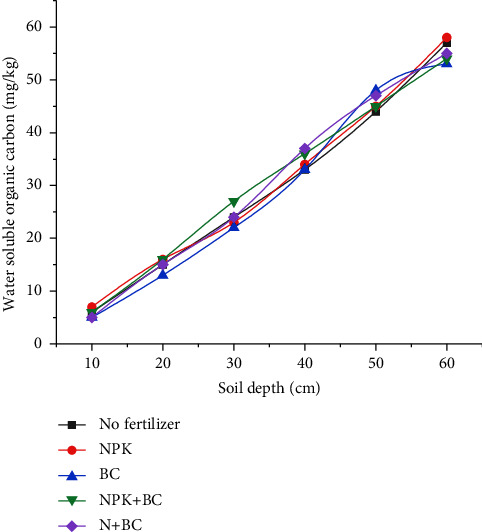
Effects of different chemical fertilizers on the content of water-soluble organic matter in the soil at 0∼60 cm depth.

The above-mentioned methods for extracting and measuring soil organic matter are used to analyze the content of water-soluble organic matter in the soil layer after the continuous application of corn biochar for six years.  Figure 5 shows the specific situation.

During the 6-year test, BC will affect the average content of water-soluble organic matter in the soil profile, whether chemical fertilizer is applied or not. In 0∼60 cm layer depth, the average content of water-soluble organic matter in soil under different fertilizers is NPK (150.5 mg/kg) > NPK + BC (132.6 mg/kg) > N + BC (123.3 mg/kg) > CK (123.0 mg/kg) > BC (116.8 mg/kg). Compared with the control group (NPK), the application of the BC fertilizer mixture results in a decrease in the content of water-soluble organic matter in the soil. Moreover, the content of water-soluble organic matter in BC treated soil is lower than that in nonfertilized soil.

### 4.2. Application of Three-Dimensional Fluorescence Spectra and Deep Learning Model in Prediction of Soil Water-Soluble Organic Matter

**Figure 6:**
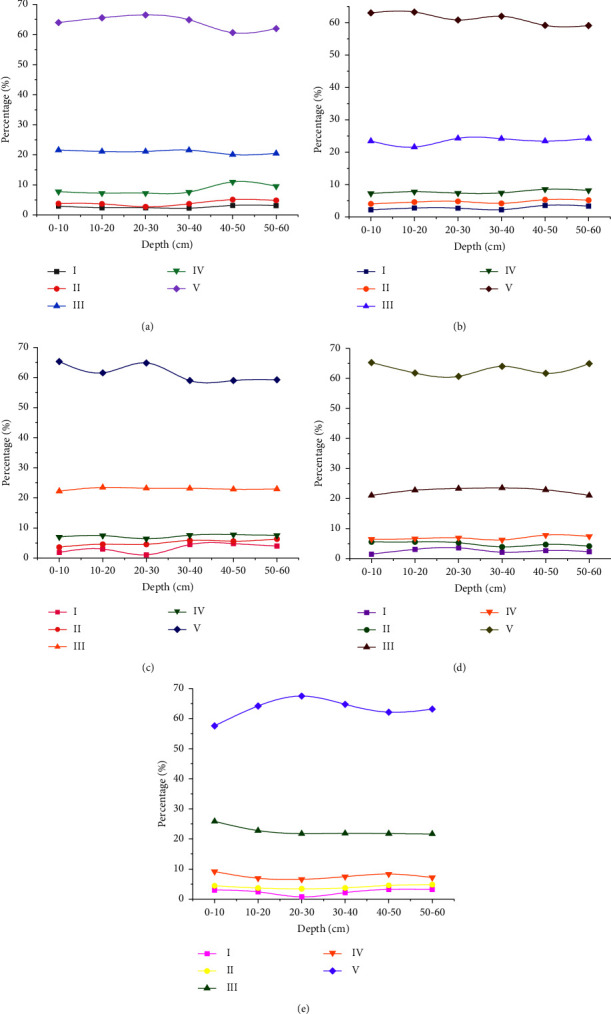
Three-dimensional fluorescence spectra of water-soluble organic matter after 6 years of fertilization (I tyrosine-like substance; II: tryptophan-like substance; III: fulvic acid-like substance; IV: soluble microbial by-products; V: humic acid-like substance): (a) no fertilizer; (b) NPK; (c) BC; (d) NPK + BC; (e) N + BC.

Three-dimensional fluorescence spectroscopy and a deep learning model are applied to detect soil water-soluble organic matter after 6 years of fertilization.  Figure 6 displays the specific results.

According to the emission and excitation wavelengths of the target molecules, the spectrometer can be divided into five parts. Section 1 (250–330 nm/200–250 nm) contains tyrosine-like substances. Section II (330–380 nm/200–250 nm) contains tryptophan-like substances. Section III (380–550 nm/220–250 nm) contains fulvic acid-like substances. Section IV (250–380 nm/250–600 nm) contains soluble microbial by-product samples. Section V (380–600 nm/250–600 nm) contains humic acid-like substances.  Figure 6 displays that the fluorescence intensity and the shape of the spectrogram change with the change in soil depth. After 6 years of fertilization, the fluorescence intensity of the third and fifth sections reaches the maximum, especially the fluorescence intensity of the fifth section, which exceeds 60%. It shows that humic acid and fulvic acid are the most important organic matter in black soil, whether or not it is improved. Compared with the BC group, BC + NPK treatment significantly increases the fluorescence intensity of section II (0–20 cm soil layer increases by 13.3%), section III (0–20 cm soil layer increases by 8.4%), and section IV (0–20 cm soil layer increases by 50.1%). The results show that BC and NPK fertilizers are enriched with tryptophan-like substances, fulvic acid-like substances, and soluble microbial by-products. Meanwhile, in the surface layer (0–20 cm), the first section (increases by 20.7%), the second section (increases by 12.2%), and the fourth section (increases by 28.4%) of BC + N fertilizer showed an increasing trend. In contrast, compared with NPK fertilizer, BC fertilizer significantly affects the fluorescence intensity of the second section (0–20 cm decreases by 9.6%) and the fourth section (0–20 cm increases by 8.5%), indicating that tryptophan and humic acid-like substances are reduced and enriched, respectively. In addition, BC + NPK fertilizer significantly increases the fourth part of soluble microbial by-product-like substances (the fluorescence intensity increases by 5.9% at 10–20 cm) and significantly reduces the first part of tyrosine-like substances (the fluorescence intensity decreases by 26.1% at 10–20 cm and 13.7% at 20–30 cm).

**Figure 7:**
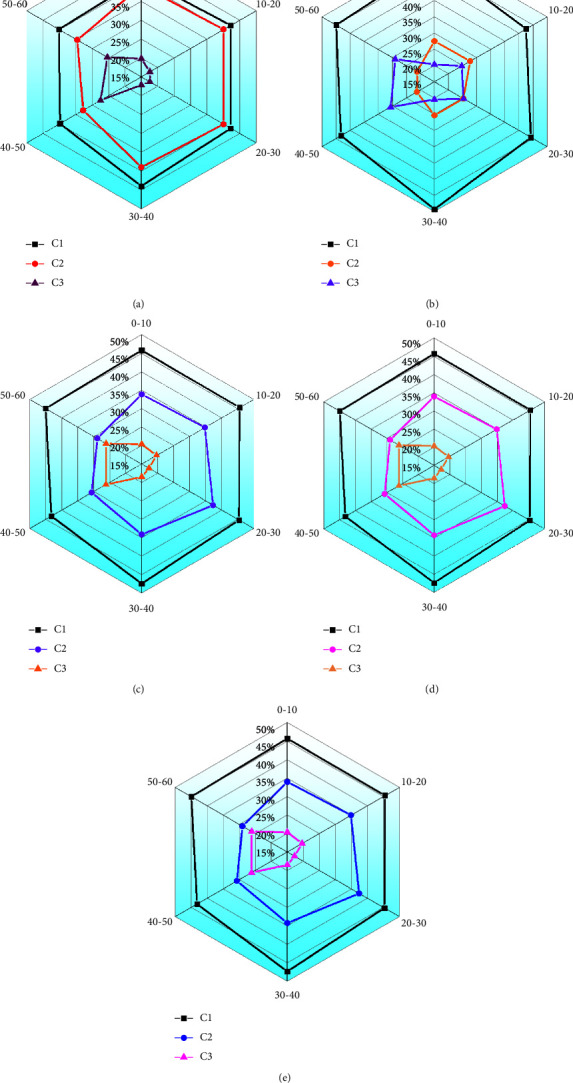
Results of different fluorescent components (C1: humic acid-like substance; C2: fulvic acid-like substance; C3: protein-like substance): (a) CK; (b) NPK; (c) BC; (d) NPK + BC; (e) N + BC.

In addition, through the model established above, different fertilizers are put into the 0–60 cm soil layer to estimate the three fluorescent components.  Figure 7 shows the specific results.

The data results of C1–C3 in Figure 7 show that the content of fulvic acid-like substance (C2) decreases and the content of protein-like substance (C3) increases with the increase in soil depth. In addition, the content of humic acid-like substance (C1) in each treatment group is higher than that of fulvic acid-like substance (C2), while the content of protein-like substance is the lowest. The protein-like substance (C3) content shows an opposite trend, while the content of C2 remains more than 50% in the deep layer (20–60 cm). It reveals that the water-soluble organic matter in the surface layer (0–20 cm) is composed of humic acids. On the contrary, the water-soluble organic matter in the subsoil layer (20–60 cm) is produced by microbial decomposition, and the level of microbial by-products increases with the increase in soil depth. In addition, after 6 years of fertilization, the level of humic acid-like substances (C1 and C2) in the subsoil layer (20–60 cm) in BC decreases by about 5% compared with NPK. The C1 content of NPK + BC treatment is the lowest and the C2 content is the highest, which is distributed in the surface layer and the lower layer.

**Figure 8:**
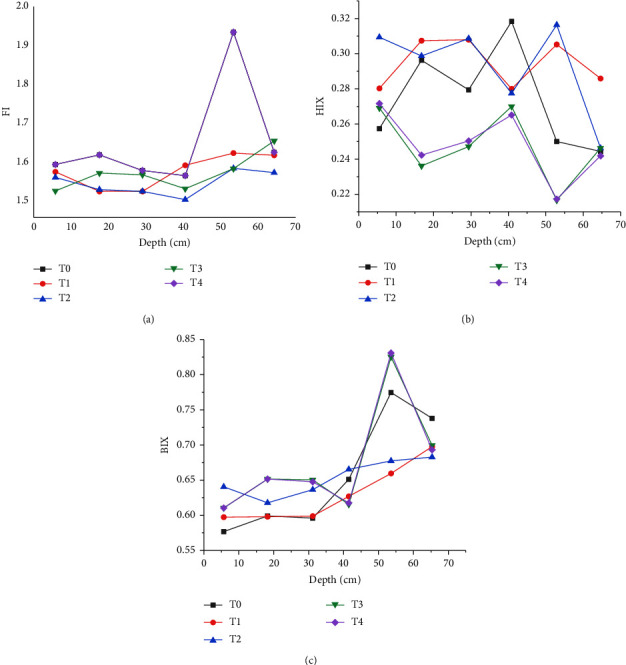
Different FIs in the whole soil depth:(a) FI; (b) HIX; (c) BIX. T0: no fertilizer; T1: NPK; T2: BC; T3: BC + NPK; T4: BC + N.

Figure 8 shows the specific results of the fluorescence index (FI), humification index (HIX) and biological index (BIX) of five treatment groups in the whole soil depth.

Figure 8 shows that FI values in different groups remain above 1.59. When BC is applied, the FI value of topsoil (0–10 cm) is the lowest, which is 1.52. When BC and NPK fertilizers are applied, the FI value of subsoil (40–50 cm) is the highest. Therefore, it can be inferred that the water-soluble organic matter imported by BC contains similar land-integrated resources in the topsoil (0–20 cm). It has moderate neogenesis characteristics and shows strong authigenic characteristics in the subsoil (20–60 cm). Most of the HIX values for different group classes are about 0.27. Compared with the control group, in which the HIX value decreases with the increase of soil depth, the average HIX value of BC-improved soil in different soil layers is higher than that of NPK fertilized soil. As expected, the HIX value of the NPK + BC group is higher than that of BC. In addition, the average HIX value of the water-soluble organic matter in surface soil and subsoil of the NPK + BC group is lower than that of the N + BC group. It shows that the contribution of NPK is greater than that of N. Besides, BI values for all groups show the same growth trend. In particular, the average value of BIX distribution in the soil profile is between 0.63 and 0.67. These results show that the content of water-soluble organic matter increases with the increase of soil depth through microbial degradation.

## 5. Conclusions

Soil quality is an important factor affecting seed growth. Based on the three-dimensional fluorescence spectra and deep learning model, this exploration studies the prediction of soil water-soluble organic matter by the continuous application of corn biochar. The results reveal the following: (1) The average content of soil water-soluble organic matter is NPK > NPK + BC > N + BC > CK > BC. (2) The application of BC for six consecutive years significantly reduces the average content of water-soluble organic matter in different soil layers, mainly because BC has a higher adsorption capacity than CK. The contents of NPK + BC and N + BC treatment groups are similar, which shows that N is the main factor affecting the soil water-soluble organic carbon level. (3) Compared with the application of BC alone, in the 0–20 cm soil layer, the second section using BC + NPK increases by 13.3%, the third section increases by 8.4%, and the fourth section increases by 50.1%. The combination of N + BC has a positive effect of 20.7%, 12.2%, and 28.4% on the fluctuation of segments I, II, and IV, respectively. (4) In the surface layer (0–20 cm), compared with BC alone, the combination of NPK + BC significantly increases the content of acid-like substances. In the black soil, with or without NPK, there is no significant difference in the level of fulvic acid-like components, especially in the surface layer (0–20 cm). However, the humification degree of T3 (NPK + BC) is slightly higher than that of T4 (N + BC) from the perspective of the surface soil decay value. In addition, the BC addition decrease in the past six years negatively impacts soil humidification. These findings are conducive to a better understanding of the dynamics of water-soluble organic matter in different soil layers under different fertilization types and the comprehensive effects of fertilizer and corn straw BC on the biogeochemical characteristics of water-soluble organic matter in black soil areas.

Although this exploration provides relatively perfect research results, only chernozem in Heilongjiang Province is taken as the research object, and the prediction of water-soluble organic matter in other types of soil has not been carried out. Besides, three-dimensional fluorescence spectroscopy and a deep learning model are used to analyze the water-soluble organic matter in soil, and other spectral techniques can be selected to improve the prediction ability of soil water solubility according to the actual situation. Meanwhile, it is essential to expand the selectivity of research samples and establish a more sound database. Therefore, the scope of research objects will be expanded, and the research methods will be optimized to provide a reference for the agricultural development of more regions in the future.

## Figures and Tables

**Table 1:** Local soil properties.

Depth (cm)	Mechanical composition (%)			Texture	Avail.^*∗*^*N*(mg/kg)	Avail.^*∗*^*P* (mg/kg)	Avail.^*∗*^*K* (mg/kg)	SOM^*∗∗*^ (g/kg)	pH	BD^*∗∗∗*^ (g/cm^3^)
	Slit	Clay	Sand							
0–30	56.3	21.9	21.8	Silty clay loam	163.3	20.61	187.92	29.87	6.74	1.31

SOM means soil organic matter; pH means potential of hydrogen; BD means bulk density.

**Table 2:** Biochar particle composition.

Particle components (%)											
Soil organic carbon (SOC)^*∗*^ (g/kg)	Ca (g/kg)	K (g/kg)	Mg (g/kg)	N (g/kg)	O (g/kg)	P (g/kg)	Si (g/kg)	pH	<0.1 mm	0.1–2 mm	>2 mm
598	3	17.0	2	7.85	166	1.327	60	8.69	15.0	60.2	24.8

## Data Availability

The data supporting the findings of the current study are available from the corresponding author upon request.
